# Odontogenic ameloblast-associated (*ODAM*) is inactivated in toothless/enamelless placental mammals and toothed whales

**DOI:** 10.1186/s12862-019-1359-6

**Published:** 2019-01-23

**Authors:** Mark S. Springer, Christopher A. Emerling, John Gatesy, Jason Randall, Matthew A. Collin, Nikolai Hecker, Michael Hiller, Frédéric Delsuc

**Affiliations:** 1 Department of Evolution, Ecology, and Organismal Biology, University of California, Riverside, CA 92521 USA; 2 Institut des Sciences de l’Évolution de Montpellier (ISEM), CNRS, IRD, EPHE, Université de Montpellier, Montpellier, France; 3 Department of Biology, Whittier College, Whittier, CA 90602 USA; 4 Division of Vertebrate Zoology and Sackler Institute for Comparative Genomics, American Museum of Natural History, New York, NY 10024 USA; 5 Max Planck Institute of Molecular Cell Biology and Genetics, Dresden, Germany; 6 Max Planck Institute for the Physics of Complex Systems, Dresden, Germany; 7 Center for Systems Biology Dresden, Dresden, Germany

**Keywords:** Edentulism, Enamel, Junctional epithelium, ODAM, Pseudogene

## Abstract

**Background:**

The gene for odontogenic ameloblast-associated (*ODAM*) is a member of the secretory calcium-binding phosphoprotein gene family. *ODAM* is primarily expressed in dental tissues including the enamel organ and the junctional epithelium, and may also have pleiotropic functions that are unrelated to teeth. Here, we leverage the power of natural selection to test competing hypotheses that *ODAM* is tooth-specific versus pleiotropic. Specifically, we compiled and screened complete protein-coding sequences, plus sequences for flanking intronic regions, for *ODAM* in 165 placental mammals to determine if this gene contains inactivating mutations in lineages that either lack teeth (baleen whales, pangolins, anteaters) or lack enamel on their teeth (aardvarks, sloths, armadillos), as would be expected if the only essential functions of *ODAM* are related to tooth development and the adhesion of the gingival junctional epithelium to the enamel tooth surface.

**Results:**

We discovered inactivating mutations in all species of placental mammals that either lack teeth or lack enamel on their teeth. A surprising result is that *ODAM* is also inactivated in a few additional lineages including all toothed whales that were examined. We hypothesize that *ODAM* inactivation is related to the simplified outer enamel surface of toothed whales. An alternate hypothesis is that *ODAM* inactivation in toothed whales may be related to altered antimicrobial functions of the junctional epithelium in aquatic habitats. Selection analyses on *ODAM* sequences revealed that the composite dN/dS value for pseudogenic branches is close to 1.0 as expected for a neutrally evolving pseudogene. DN/dS values on transitional branches were used to estimate *ODAM* inactivation times. In the case of pangolins, *ODAM* was inactivated ~ 65 million years ago, which is older than the oldest pangolin fossil (*Eomanis*, 47 Ma) and suggests an even more ancient loss or simplification of teeth in this lineage.

**Conclusion:**

Our results validate the hypothesis that the only essential functions of *ODAM* that are maintained by natural selection are related to tooth development and/or the maintenance of a healthy junctional epithelium that attaches to the enamel surface of teeth.

**Electronic supplementary material:**

The online version of this article (10.1186/s12862-019-1359-6) contains supplementary material, which is available to authorized users.

## Background

The secretory calcium-binding phosphoprotein (SCPP) gene family contains 23 members in the human genome and achieved its present diversity through an extensive series of gene duplication events [1, 2]. In mammals, different SCPP genes are expressed in mineralized tissues, mammary glands, and salivary glands [2]. Among the genes that are expressed in mineralized tissues of vertebrates, some are important for bone and/or dentin formation whereas others are critical for the synthesis of enamel (tetrapods) and/or enameloid (bony fishes) [2]. The gene that encodes odontogenic ameloblast-associated (*ODAM*) is a member of the SCPP family and is thought to have originated as far back as the common ancestor of tetrapods and bony fishes more than 450 million years ago [2–4]. Early in tooth development, *ODAM* is highly expressed in the enamel organ that gives rise to ameloblasts that produce enamel. Later in tooth development, *ODAM* is expressed in the junctional epithelium, which is a specialized epithelium that attaches the soft tissue of the gingiva (gums) to the enamel surface of the tooth and protects against bacteria that promote periodontal disease [5–9]. In this capacity, ODAM plays a critical role in the first line of defense against bacterial invasion [10]. ODAM has also been shown to upregulate the expression of the *MMP20* gene [11], which encodes a matrix metalloproteinase required for proper enamel development, and to promote the nucleation of hydroxyapatite crystals [12]. Young *ODAM* knockout mice show no abnormalities in enamel volume, density, and organization, but in older individuals the junctional epithelium shows significant detachment and apical downgrowth [13]. Beyond its expression in ameloblasts and the junctional epithelium, *ODAM* is expressed in many non-dental tissues in humans [8, 14]. Of these, expression levels are strongest in salivary glands, mammary glands, and the trachea [8]. *ODAM* is also expressed in carcinomas of the stomach, breast, lung, and ovary [14, 15].

Previous studies have shown that one or more of nine tooth-related genes (*ACP4, AMBN, AMEL, AMTN, C4orf26* (= *ODAPH*)*, DSPP, ENAM, KLK4, MMP20*) are inactivated in a variety of toothless vertebrates that have been investigated including birds [16–19], turtles [20], and several mammalian lineages comprising baleen whales, pangolins, anteaters, and Steller’s sea cow [19, 21–28]. Many of these genes are also inactivated in mammals with enamelless teeth such as pygmy and dwarf sperm whales, aardvarks, sloths, and armadillos [19, 23, 24, 28–30]. The widespread inactivation of these genes in edentulous and enamelless vertebrates suggests that their only essential functions are related to tooth development even if they are sometimes expressed in non-dental tissues [19].

Given its important role in amelogenesis, *ODAM* has emerged as yet another gene that should be screened in toothless and enamelless vertebrates for evidence of inactivating mutations. If *ODAM* is consistently inactivated in vertebrates that lack teeth or the outer enamel covering on their teeth, then *ODAM*’s only essential functions would appear to be tooth-related even though *ODAM* is expressed in other tissues [14]. By contrast, if *ODAM* gene sequences are intact in toothless and enamelless vertebrates this would provide evidence for pleiotropic, non-dental functions that affect fitness and are protected by natural selection.

Here, we test the hypothesis that the main function of *ODAM* is related to enamel formation by investigating whether this gene remains under purifying selection in placental mammals that have lost their teeth or the outer enamel covering on their teeth. To this end, we compiled and screened complete protein coding sequences of the *ODAM* gene for inactivating mutations in 165 placental mammal species comprising representatives of all extant placental orders. In support of our hypothesis, we found that all lineages without teeth (baleen whales, pangolins, anteaters) or without enamel on their teeth (aardvark, sloths, armadillos) have inactivated *ODAM*. Finally, selection analyses to estimate when purifying selection on *ODAM* was relaxed in lineages with inactivated *ODAM* provide new insights into when teeth or their outer enamel caps were lost in these lineages.

## Methods

### Taxon sampling

Taxon sampling included 165 placental mammal species, of which 135 are from assembled genomes in public databases, 13 are from unassembled sequence reads in the Sequence Read Archive (SRA) or European Nucleotide Archive (ENA), eight are from a combination of assembled genomes and unassembled sequence reads, and nine are from new Illumina HiSeq2500, HiSeq4000, HiSeqX10, or NovaSeq data (Additional file 1: Table S1). Taxon sampling included 12 taxa from Afrotheria, ten taxa from Xenarthra, 61 taxa from Euarchontoglires, and 82 taxa from Laurasiatheria. Among the afrotherians, three taxa (*Mammut americanum* [American mastodon], *Mammuthus primigenius* [woolly mammoth], *Palaeoloxodon antiquus* [straight-tusked elephant]) are extinct. Toothless taxa included two vermilinguans (anteaters), two pholidotans (pangolins), and seven mysticetes (baleen whales); taxa that lack enamel on their teeth included one tubulidentate (aardvark), two folivorans (sloths), and six cingulatans (armadillos).

### BLAST searches and alignments

Genomic sequences encoding *ODAM* were downloaded from NCBI for representatives of Euarchontoglires (*Homo sapiens*), Laurasiatheria (*Bos taurus*), and Afrotheria (*Trichechus manatus*). These sequences were employed in BLAST searches against other placental mammals in NCBI’s 'RefSeq Genome' and 'Whole-genome shotgun contigs' databases. We used megablast for highly similar sequences and the more sensitive blastn for less similar sequences when megablast searches failed to return results. Complete coding sequences and intervening introns were imported into Geneious 11.1.5 [31] and aligned with MAFFT [32, 33] with minor adjustments by eye. For five xenarthrans (*Cabassous unicinctus*, *Tolypeutes matacus*, *Euphractus sexcinctus*, *Choloepus didactylus*, *Tamandua tetradactyla*), we imported Discovar de novo (https://software.broadinstitute.org/software/discovar/blog/) genome assemblies into Geneious [12] and performed local BLAST searches (discontiguous megablast) using reference xenarthran sequences (*Dasypus novemcinctus*, *Choloepus hoffmanni*). With a few exceptions (see below), exon-intron boundaries are conserved among placental mammals. In cases where there were suspected assembly gaps with missing exons, we used sequences from the closest available taxon (exon plus ~ 100 bp of each flanking intron) to perform additional BLAST searches with megablast against the Sequence Read Archive (SRA). Sequences from SRA BLAST searches were imported into Geneious and mapped to the closest reference sequence to assemble missing exons and their flanking intron sequences. A similar strategy was employed to retrieve complete protein-coding sequences for *ODAM* in taxa with unassembled genomes in the SRA. We also used a mapping to reference approach in Geneious to retrieve *ODAM* sequences from unpublished Illumina data for *Choeropsis liberiensis* (pygmy hippopotamus) and three xenarthrans. For *C. liberiensis*, we mapped reads to the *ODAM* sequence for *Hippopotamus amphibius* (river hippopotamus). For the three xenarthrans (*Chlamyphorus truncatus*, *Calyptophractus retusus*, *Cyclopes didactylus*), we imported short reads into Geneious and mapped them to various xenarthran reference sequences. Individual exon sequences (and flanking introns) from each SRA taxon were concatenated into a single sequence with Geneious and aligned to the global alignment.

### Inactivating mutations

We inspected the final alignment (165 taxa) for inactivating mutations including exon deletions, frameshift mutations, altered start and stop codons, premature stop codons, and splice site mutations. We also used the alignment program MACSE v2 [34, 35] to crosscheck the results of manual inspections for frameshifts and premature stop codons in coding sequences. Parsimony optimizations with delayed transformation (deltran) were performed with PAUP* 4.0a150 [36] to map inactivating mutations to branches of the phylogeny of placental mammals.

### Phylogenetic analyses

RAxML 8.2.10 [37, 38] on CIPRES [39] was used to estimate a maximum likelihood tree for the complete protein-coding sequence alignment. Rapid bootstrap analysis (500 pseudoreplications) [40] and a search for the best tree were performed in a single run. These analyses were performed with a GTR + Γ model of sequence evolution.

### Selection analyses

We first reconstructed the evolution of the dN/dS ratio of *ODAM* across the placental mammal phylogeny using the Bayesian approach implemented in Coevol 1.4b [41] on the complete alignment of 165 sequences containing 279 aligned codons. We used the dsom estimation procedure that jointly estimates branch specific dN/dS ratios, divergence times, body sizes, generation times, and ages at sexual maturity modeled as a multivariate Brownian diffusion process [42]. DN/dS analyses with Coevol were performed to detect and visualize patterns of dN/dS variation across a tree. The assumed topology was the *ODAM* gene tree after making several rearrangements to correct for discrepancies between the gene tree and species tree [43–46] as follows: *Chaetophractus* sister to other Chlamyphoridae; *Orycteropus* sister to Afrosoricida + Macroscelidea; *Solenodon* sister to other Eulipotyphla; *Sus* sister to Ruminantia + Whippomorpha; *Physeter* sister to other Odontoceti; Pholidota sister to Carnivora; *Rhinolophus* + *Hipposideros* sister to *Megaderma*; and *Nomascus* sister to Hominidae. We assumed fossil calibrations from previous studies [44, 46, 47] and extracted life history traits from PanTHERIA [48]. We set the prior on the root node to 97 Ma with a standard deviation of 20 Ma following the dating of Meredith et al. [44]. We ran two independent MCMC for a total of 5000 cycles sampling parameters every cycle. After checking for convergence by monitoring the effective sample size of the different parameters using the tracecomp command, we excluded the first 500 points of each MCMC as burnin, and made inferences from the remaining 4500 sampled points of each chain.

Additional dN/dS analyses were performed with the codeml program of PAML 4.4 [49]. Codeml does not employ the autocorrelative model of Brownian motion [50] that is incorporated into Coevol. For computational efficiency and tractability, codeml analyses were performed with 96 taxa after pruning all but one representative for mammalian families with *ODAM* sequences that are intact for all exemplars included in our study. We used 15 dN/dS categories based on reconstructions of ancestral states for tooth/enamel loss [23, 24] and the occurrence of frameshift mutations, premature stop codons, and/or exon deletions in independent lineages. We ignored taxa (*Hyaena hyaena, Elaphurus davidianus, Eulemur flavifrons* [see below]) that contained just a single splice site mutation except for *Physeter macrocephalus.* This taxon is of interest because the next possible donor splice site (GT) for intron 4 is 75 bp downstream from the canonical splice site and has a very low MaxEntScan score of − 17 (http://genes.mit.edu/burgelab/maxent/Xmaxentscan_scoreseq.html [51]). (For reference, splice sites are predicted to be functional when they have MaxEntScan scores of > 3 [52]). Moreover, all other toothed whales have one or more frameshift mutations and/or premature stop codons in *ODAM*. The 15 categories that we employed in dN/dS analyses are as follows: one category for branches leading to intact sequences that lack inactivating mutations; one category for pseudogenic branches that postdate tooth or enamel loss (parsimony deltran optimization) and/or the occurrence of an inactivating mutation on an earlier branch; nine categories for transitional branches that record the first inactivating mutation in *ODAM* (*Dasypus novemcinctus*; stem Chlamyphoridae; *Orycteropus afer*; stem Pholidota; *Neomonachus schauinslandi*; *Lipotes vexillifer*; stem to Delphinidae + Monodontidae + Phocoenidae; *Physeter macrocephalus; Callithrix jacchus*); one category for the stem Pilosa branch where enamel was lost [23]; one category for the stem Mysticeti branch where teeth were lost [24]; one category for the stem branch leading to proboscideans with missing exon 6; and one category for proboscidean branches that post-date the loss of exon 6. We employed the same topological corrections for the species tree as detailed above for the Coevol analysis. Codeml analyses were performed with two different codon frequency models, CF1 and CF2. Codon frequencies are estimated from mean nucleotide frequencies across all three codon positions in CF1 and from mean nucleotide frequencies at each of the individual codon positions in CF2. All frameshift insertions were removed from the alignment prior to codeml analyses, as were in-frame insertions that are unique to one or a few taxa. Also, premature stop codons were recoded as missing data. The final alignment for codeml analyses contained 837 aligned positions (279 codons).

### Estimation of ODAM inactivation times

We used equations from Meredith et al. [23] to estimate when *ODAM* was inactivated in different placental lineages. Calculations were performed with dN/dS values that were obtained using two different codon models in codeml (CF1, CF2), fixed (1.0) versus estimated values for the dN/dS value on fully pseudogenic branches, and equations that allow for one versus two synonymous substitution rates [23]. Divergence times for relevant nodes in these calculations were taken from Springer et al. [53] for *Callithrix* to *Aotus*, Delsuc et al. [45] for all divergences in Xenarthra, Gaubert et al. [54] for *Manis pentadactyla* to *M. javanica*, and Foley et al. [46] for all other divergence dates.

### Screen for convergently inactivated genes in edentulous and enamelless mammals

We performed a genomic screen for convergently inactivated genes based on a Forward Genomics approach with a multispecies genome alignment [28]. Briefly, this screen is based on the percentage of the reading frame of the protein-coding gene that is intact and is calculated based on the relative position of inactivating mutations and the partial and/or complete loss of entire exons. This percentage is hereafter denoted as %intact. We considered 61 placental mammals for which inactivating mutations have been computed previously [28]. These species include the edentulous *Balaenoptera acutorostrata scammoni* and *Manis pentadactyla* and the enamelless *Orycteropus afer* and *Dasypus novemcinctus*, representing four independent lineages. In contrast to a previous screen [28], we performed a more sensitive search by classifying a gene as *inactivated* if %intact is < 80% and classifying a gene as *intact* if %intact is ≥90%. We then screened for genes that are *inactivated* in at least two of the four edentulous or enamelless species, but are *intact* in at least 90% of species that have tooth enamel. The resulting candidate list of the screen was then sorted (largest to smallest) by the number of species without teeth or enamel that exhibit an *inactivated* gene (%intact < 80%) and, in case of a tie, sorted (smallest to largest) by the number of species with tooth enamel that do not have an *intact* version of the gene (%intact < 90%). Finally, we used a phylogenetic generalized least squares approach [55] to compute a phylogeny-corrected *P*-value for the association between %intact and the presence/absence of enamel.

## Results

### Multiple alignment and phylogenetic analysis

**Fig. 1:**
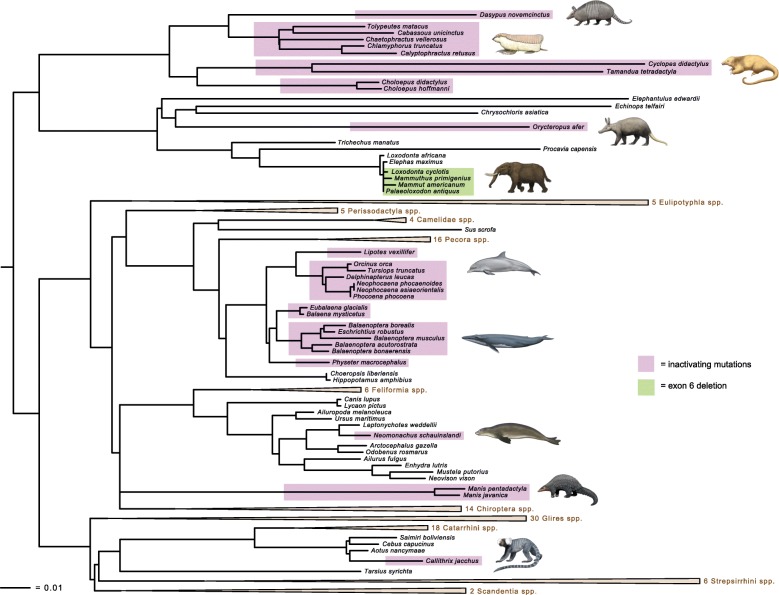
Maximum likelihood phylogram (ln L = − 27,926.663886) based on *ODAM* protein-coding sequences for 165 placental mammal species. Pink boxes indicate taxa with inactivating mutations in *ODAM*; green boxes indicate proboscideans that are missing exon 6 of *ODAM*. The tree is rooted between Atlantogenata and Boreoeutheria. Some clades for which all of the constituent taxa have intact *ODAM* sequences were collapsed, and the length of the Glires clade branch was halved to improve aesthetics (see TreeBASE [http://purl.org/phylo/treebase/phylows/study/TB2:S23531] for ML phylogram with all 165 taxa)

The 165-taxon alignment for the complete protein-coding sequence of *ODAM* is 1177 nucleotides including frameshift insertions and is available at TreeBASE (http://purl.org/phylo/treebase/phylows/study/TB2:S23531). The shape parameter of the gamma distribution was estimated at 1.98 by RAxML. Figure 1 shows the maximum likelihood gene tree (ln L = − 27,926.663886). This gene tree was not rooted with a marsupial, but is consistent with the monophyly of the four major clades of placental mammals (Xenarthra, Afrotheria, Laurasiatheria, Euarchontoglires) [56–64]. Other superordinal groups that were recovered on the *ODAM* gene tree include Afroinsectiphilia, Paenungulata, Variamana, Euungulata, Glires, and Euarchonta. Thirteen of 19 placental orders included more than one species and in all cases these orders were recovered as monophyletic. Several features of the *ODAM* gene tree are in minor conflict with generally accepted species trees including the placement of *Physeter* as the sister group to mysticetes rather than other odontocetes [65, 66], an association of Pholidota with Chiroptera rather than Carnivora [44], a sister group relationship between Hipposideridae + Rhinolophidae and yangochiropteran bats instead of with other rhinolophoid bats [43, 67], an association of chlamyphorine armadillos (*Chlamyphorus, Calyptophractus*) with *Chaetophractus* rather than with tolypeutines (*Tolypeutes, Cabassous*) [45], a basal split between *Condylura* and other eulipotyphlans instead of between *Solenodon* and other eulipotyphlans [44, 68, 69], and the nonmonophyly of Elephantidae with the mammutid *Mammut americanum* nested inside of this clade instead of sister to this clade [70, 71].

### Inactivating mutations

**Fig. 2:**
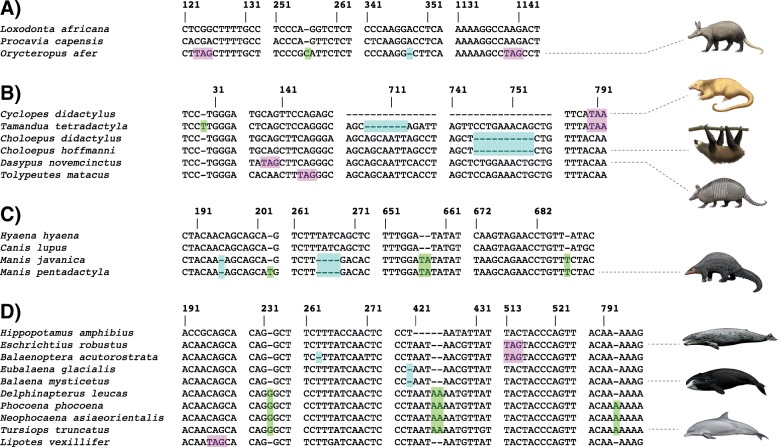
Examples of inactivating mutations in *ODAM*. **a**) *Orycteropus afer* (aardvark). **b**) Xenarthra (anteaters, sloths, armadillos). **c**) *Manis* spp. (pangolins). **d**) Cetacea (whales, dolphins, porpoises). Green boxes denote frameshift insertions; blue boxes denote frameshift deletions; and purple boxes denote premature stop codons. *Loxodonta*, *Procavia*, *Hyaena*, *Canis*, and *Hippopotamus* have intact *ODAM* sequences and were included in different alignment panels to provide context for inactivating mutations

One or more inactivating mutations (frameshifts, altered start and stop codons, premature stop codons, splice site mutations) were discovered in all placental taxa without teeth or without enamel on their teeth. Table 1 provides a complete list of inactivating mutations in these taxa and examples of inactivating mutations are shown in Fig. 2. Shared inactivating mutations were discovered in Vermilingua, *Choloepus*, Chlamyphoridae, Tolypeutinae, *Manis*, Balaenidae, Balaenopteroidea, and in the two minke whales (*Balaenoptera acutorostrata* and *B. bonaerensis*). Among edentulous forms, taxa with the largest number of inactivating mutations are *Manis javanica* (15), *Manis pentadactyla* (14), and *Tamandua tetradactyla* (14), whereas the fewest inactivating mutations are found in *Balaena mysticetus* (1) and *Balaenoptera musculus* (1). Among taxa with enamelless teeth, *Orycteropus afer* and *Tolypeutes matacus* each have four inactivating mutations whereas *Choloepus didactylus* only has one inactivating mutation. However, it should be noted that only 8 of 10 exons had BLAST results for *C. didactylus*. An association between *ODAM* inactivation and the loss of teeth or enamel is further supported by a genomic screen for genes that have inactivating mutations and an abolished reading frame in edentulous and enamelless taxa, which uncovered *ODAM* at rank 7 together with other tooth-related genes (Additional file 2: Table S2).

**Table 1:** Inactivating mutations and whole exon deletions in the *ODAM* gene of toothless, enamelless, and other placental mammals

Taxon	Exon 1	Exon 2	Exon 3	Exon 4	Exon 5	Exon 6	Exon 7	Exon 8	Exon 9	Exon 10	Splice site mutations^a^
*Orycteropus*			123-125S	256I, 348D					1081I	1139-1141S	In3Do (AT)
Vermilingua									790-792S		
*Tamandua tetradactyla*	29I		105D	306-308S, 336I, 374-375D	430D	546-548S, 573-575S	668-670S	707-713D	797-799S, 1103-1112D		In2Do (AT)
*Cyclopes didactylus*			127D				671I	NRM		NRM	In1Ac (AT), In4Do (GG), In6Do (AT), In7Do (AT)
*Choloepus*								745-754D			
*Choloepus hoffmanni*				400-402S							
*Choloepus didactylus*		NBR								NBR	
*Dasypus novemcinctus*			138-140S	400-402S		606-608S			1127-1129S		
Chlamyphoridae		93-98D^b^									
*Chaetophractus vellerosus*							672-674S		820D		In9Ac (CG)
*Cabassous* + *Calyptophractus*											In4Ac (TG)^c^
Tolypeutinae			144-146S			570-573D					In6Ac (AA), In9Ac (AA)
*Tolypeutes matacus*	1-3SCD				NBR		672-674S				In8Do (TT)
*Cabassous unicinctus*						595-598D		NBR	NBR		
*Chlamyphorus truncatus*	NRM	NRM	NRM	NRM	NRM		672-674S				In8Do (RT)
*Calyptophractus retusus*									1092D		
*Manis*				195D, 265-268D, 345D		518D, 561D	657-658I, 672-674S	687I	1088D		In4Do (AT), In5Do (CT), In6Do (CT)
*Manis pentadactyla*			123-125S	203I							
*Manis javanica*				350D					851-1059I		In9Do (AC)
Balaenidae				420D							
*Eubalaena glacialis*											In6Do (AT)
Balaenopteroidea											In2Do (AT)
*Eschrichtius robustus* + *Balaenoptera acutorostrata* + *B. bonaerensis* + *B. borealis*						513-515S					
*Balaenoptera acutorostrata* + *B. bonaerensis*				263D							
*Balaenoptera bonaerensis*				382-384S							
*Balaenoptera borealis*										1159-1165D^d^	
*Physeter macrocephalus*											In4Do (AT)
*Lipotes vexillifer*				195-197S, 264-266S, 288-290S							
Delphinidae + Monodontidae + Phocoenidae				232I, 424-425I							
Phocoenidae									793I		
*Phocoena phocoena*											In2Ac (AT)
*Tursiops truncatus*									793I		
*Neomonachus schauninslandi*			104-105D	198-200S	429-479D						In2Ac (AA)
*Callithrix jacchus*					477-479S						In4Ac (AC)

^a^Mutated splice sites are shown in parentheses

^b^Deletion includes last six bp of exon 2 and first nine bp of intron 2 including donor splice site

^c^No blast results for *Chlamyphorus* and *Tolypeutes*

^d^Eight bp deletion that includes stop codon

Abbreviations: *Ac* acceptor splice site, *D* frameshift deletion, *Do* donor splice site, *I* frameshift insertion, *In* intron, *NBR* no blast results, *NRM* no reads mapped, *S* premature stop codon relative to original reading frame, *SCM* start codon mutation

Position numbers correspond to the complete protein-coding alignment for 165 placental mammals

In addition to inactivating mutations in edentulous and enamelless taxa, we also discovered inactivating mutations in all eight representatives of Odontoceti including inactivating mutations that are shared by members of the clade Delphinoidea (Phocoenidae [3 spp.], Monodontidae [1 sp.], and Delphinidae [2 spp.]). The number of inactivating mutations among different odontocete species ranges from one in *Physeter macrocephalus* (splice site mutation) to four in the three phocoenid species (*Phocoena phocoena*, *Neophocaena phocaenoides*, *N. asiaeorientalis*).

Beyond these odontocetes, additional taxa with enamel-capped teeth had putative inactivating mutations. The phocid (seal) *Neomonachus schauinslandi* has one 2-bp frameshift deletion, one premature stop codon, an acceptor splice site mutation (intron 2 acceptor, AG = > AA), and a putative deletion of exon 5. However, genomic Illumina data are not available for *Neomonachus,* and we were unable to validate these mutations. By contrast, the New World primate *Callithrix jacchus* has a premature stop codon in exon 5 and an acceptor splice site mutation (AC) in intron 4, both of which are confirmed by SRA data. The mustelid *Enhydra lutris* has a 1-bp frameshift insertion in exon 1. Here, Illumina data for three individuals of *Enhydra* (SRR5768046, SRR5768052, SRR6450476) suggest that this deletion either has allelic variation or that there is an additional paralog of exon 1 in the genome of this species. Finally, there are splice site mutations (relative to canonical splice sites) in the strepsirrhine primate *Eulemur flavifrons* (intron 7 acceptor, AG = > TT), in the carnivoran *Hyaena hyaena* (intron 8 donor, GT = > TT), and in the deer *Elaphurus davidianus* (intron 5 donor, GT = > CT). However, all of these splice site mutations can be accommodated by alternative splice sites that result in slightly shorter exons (3 bp shorter in *Elaphurus*, 6 bp shorter in *Hyaena*, 18 bp shorter in *Eulemur*), and such evolutionary splice site shifts provide no indication for gene loss [72].

**Fig. 3:**
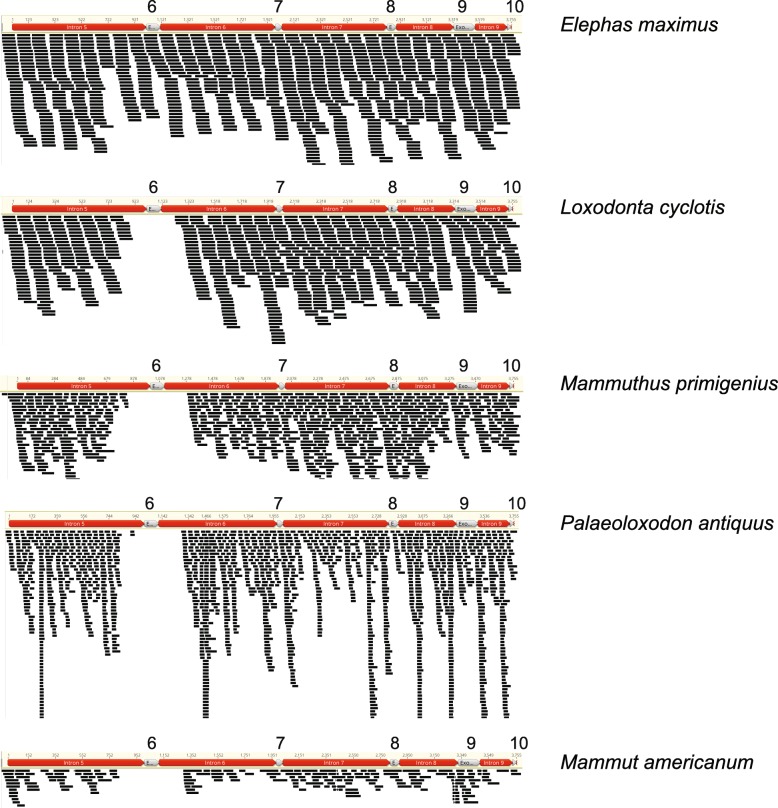
Map to reference coverage of Illumina reads onto a contiguous block of *Loxodonta africana* (African savannah elephant) *ODAM* that begins with intron 5 and ends with exon 10. Map to reference results suggest that exon 6 is retained in *Elephas maximus* (Asian elephant) but is missing in several other proboscideans (*Loxodonta cyclotis* [African forest elephant]*, Mammuthus primigenius* [woolly mammoth]*, Palaeoloxodon antiquus* [straight-tusked elephant]*, Mammut americanum* [American mastodon])

Finally, there were no BLAST results for exon 6 of *ODAM* in three elephantids (*Mammuthus primigenius, Palaeoloxodon antiquus, Loxodonta cyclotis*) and the mammutid *Mammut americanum* (Fig. 3). By contrast, this exon is present in the elephantids *Loxodonta africana* and *Elephas maximus*.

### Selection analyses

**Fig. 4:**
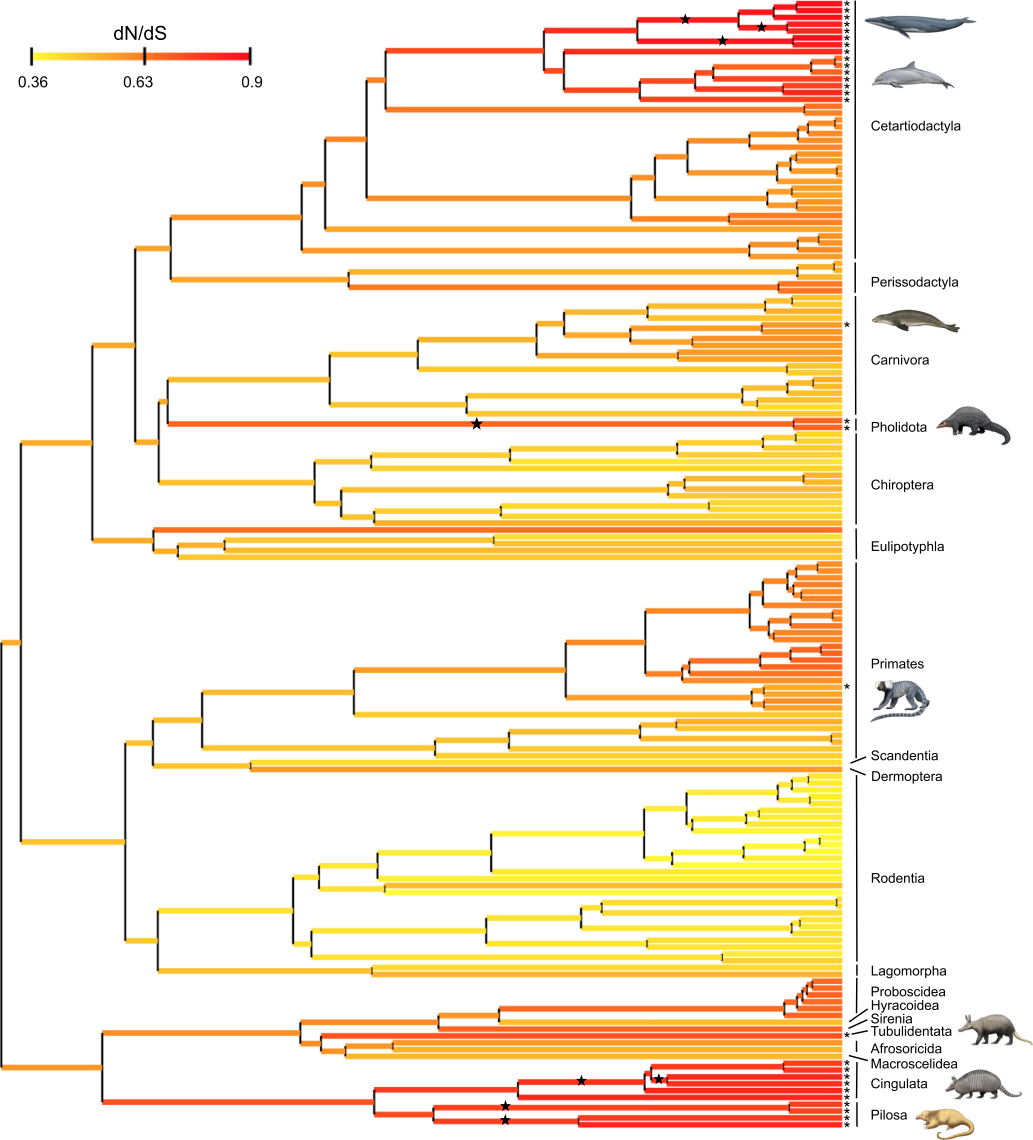
Bayesian reconstruction of dN/dS for 165 *ODAM* sequences across the placental phylogeny. The variation of dN/dS was jointly reconstructed with divergence times while controlling the effect of three life-history traits (body mass, longevity, and maturity). Asterisks at the tips of terminal branches indicate non-functional sequences (pseudogenes). Stars indicate branches on which shared inactivating mutations were inferred in toothless or enamelless clades. The tree is rooted between Atlantogenata and Boreoeutheria. Placental orders are delimited to the right of species tree tips

The joint Bayesian reconstruction of dN/dS across the placental phylogeny highlights large variation of selection pressure among lineages. Mean dN/dS values for functional branches range from 0.36 in the kangaroo rat *Dipodomys ordii* to 0.70 in the bonobo *Pan paniscus* and are compatible with purifying selection (Fig. 4). At the ordinal level, rodents and bats show the lowest average dN/dS values of 0.41 and 0.48, respectively, whereas functional cetartiodactyl and primate sequences exhibit larger mean values of 0.59 and 0.60, respectively. These results illustrate the influence of different life-history traits. A consequence of relaxed selection constraints is that the non-functional sequences show elevated dN/dS values for pilosans (0.79), cingulatans (0.81), aardvark (0.73), pangolins (0.70), odontocetes (0.75) and mysticetes (0.87). However, the Hawaiian monk seal (*Neomonachus schauinslandi*) and the common marmoset (*Callithrix jacchus*), which present inactivating mutations in their *ODAM* sequences, show only moderately elevated dN/dS values of 0.58 and 0.54, respectively. Transitional branches where enamel loss was inferred based on ancestral reconstructions [23, 24] all have elevated dN/dS values: stem Pilosa (0.75), stem Chlamyphoridae (0.81), stem Pholidota (0.70), and stem Mysticeti (0.82).

The results of selection (dN/dS) analyses using codeml are summarized in Table 2. The dN/dS values for functional branches that lead to taxa with intact protein-coding sequences for *ODAM* and enamel-capped teeth are ~ 0.49 and ~ 0.52 with codon frequency model 1 (CF1) and codon frequency model 2 (CF2), respectively, confirming that *ODAM* has evolved overall under purifying selection. By contrast, the values for pseudogenic branches that post-date the first occurrence of an inactivating mutation(s) on an earlier branch are ~ 1.04 (CF1) and 1.11 (CF2). These values are close to the expected value of 1.00 for neutrally evolving pseudogene sequences. Transitional branches where enamel was lost based on ancestral reconstructions [23, 24] include stem Pilosa, stem Pholidota, stem Mysticeti, and *Orycteropus*. DN/dS values for these branches are ~ 0.12 (CF1) to ~ 0.14 (CF2) for stem Pilosa, ~ 0.93 (CF1) to ~ 1.0 (CF2) for stem Pholidota, and ~ 0.88 (CF1) to ~ 0.93 (CF2) for *Orycteropus*. The codeml value for Mysticeti is based on only two nucleotide substitutions, both of which are nonsynonymous, leading to parameter unidentifiability. Other transitional branches for enamelless species based on the occurrence of the first inactivating mutation(s) include stem Chlamyphoridae and *Dasypus.* The dN/dS values for stem Chlamyphoridae are ~ 1.01 (CF1) and ~ 1.10 (CF2), whereas those for *Dasypus* are ~ 0.96 (CF1) and ~ 1.02 (CF2).

**Table 2:** Results of selection (dN/dS=ω) analyses with codon frequency models 1 (CF1) and 2 (CF2) and 15 different branch categories

Branch category	CF1			CF2		
	dN/dS	N * dN	S * dS	dN/dS	N * dN	S * dS
Background	0.4904	2230.2	1906.8	0.5202	2203.6	2015.4
Pseudogenic	1.0446	333.7	133.9	1.1060	332.0	142.6
*Orycteropus afer*	0.8843	67.9	32.2	0.9340	66.9	34.1
Stem Pilosa	0.1227	2.2	7.6	0.1443	2.4	8.1
*Dasypus novemcinctus*	0.9609	46.3	20.2	1.0190	46.5	21.7
Stem Chlamyphoridae	1.0083	9.2	3.8	1.1043	9.2	4.0
Stem Pholidota	0.9333	60.7	27.2	0.9980	59.8	28.5
Stem Mysticeti	999.0000	2.0	0.0	999.0000	2.0	0.0
*Physeter macrocephalus*	0.9000	11.7	5.4	1.0071	11.7	5.5
*Lipotes vexillifer*	1.1064	11.0	4.2	1.1690	10.9	4.4
Stem to Delphinidae + Monodontidae + Phocoenidae	999.0000	7.7	0.0	999.0000	7.5	0.0
*Callithrix jacchus*	0.8450	13.3	6.6	0.8965	13.1	7.0
*Neomonachus schauinslandi*	0.4035	3.9	4.0	0.4072	3.6	4.2
Stem to *Mammut americanum + Loxodonta cyclotis + Palaeoloxodon antiquus + Mammuthius primigenius*	0.7350	0.0	0.0	0.0001	0.0	0.0
Proboscidean branches that post-date loss of exon 6	0.2133	2.4	4.6	0.2287	2.3	4.8

Symbols: * is multiplication symbol

Transitional branches that exhibit the first inactivating mutation(s) among odontocetes all have high dN/dS values including *Physeter* (~ 0.90 [CF1], ~ 1.01 [CF2]), and *Lipotes* (~ 1.11 [CF1], ~ 1.17 [CF2]). The dN/dS value for the stem branch leading to Delphinidae + Phocoenidae + Monodontidae is based on 7.5–7.7 substitutions, all of which are nonsynonymous, and is undefined. Other transitional branches with putative inactivating mutations are *Callithrix*, *Neomonachus,* and the stem branch leading to three elephantids + *Mammut americanum*. *Callithrix* has an elevated dN/dS value (~ 0.85 [CF1], ~ 0.90 [CF2]). *Neomonachus* has dN/dS values of ~ 0.40 (CF1) to ~ 0.41 (CF2) that are below the background ratios for codeml. Finally, there are no inferred substitutions on the stem branch leading to proboscideans that are missing exon 6 so the codeml values for this branch can be ignored. The dN/dS values for crown proboscidean branches that are missing exon 6 are ~ 0.21 (CF1) and ~ 0.23 (CF2) and are below the background ratios of ~ 0.49 and ~ 0.52, respectively.

### Inactivation times

**Fig. 5:**
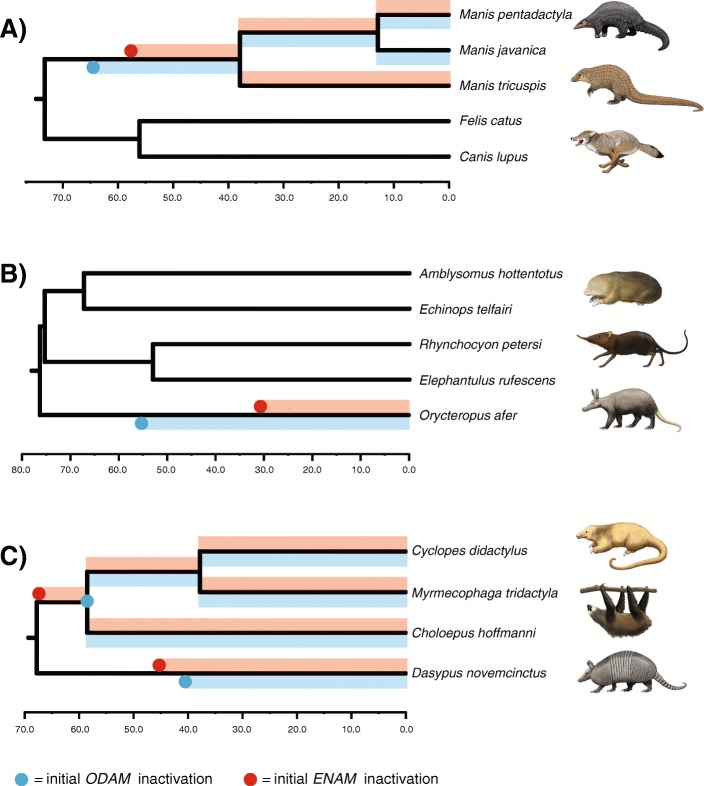
Estimated inactivation times in *ENAM* versus *ODAM*. **a**) *Manis* spp. (pangolins). **b**) *Orycteropus afer* (aardvark). **c**) Pilosa (anteaters, sloths) and *Dasypus novemcinctus* (nine-banded armadillo). Inactivation dates are mean values based on eight different combinations of two different codon models in codeml (CF1, CF2), fixed (1.0) versus estimated values for the dN/dS value on fully pseudogenic branches, and equations that permit for one versus two synonymous substitution rates [23]. *Manis tricuspis* is missing from the *ODAM* data set and *M. javanica* was not included in Meredith et al.’s [23] *ENAM* data set

Estimates of inactivation times for *ODAM* based on dN/dS ratios and equations in Meredith et al. [23] are provided in Table 3 for several transitional branches. The mean estimate for the inactivation of *ODAM* on the stem Pholidota branch is 64.15 Ma (range = 73.04–57.34 Ma) (Fig. 5). The mean inactivation estimate for *Orycteropus ODAM,* in turn, is 55.63 Ma (range 58.9–48.2 Ma). Among cingulatans, the mean inactivation time in *Dasypus* is 40.43 Ma (range 45.45–36.38 Ma) and is similar to the mean estimate of 45.29 Ma for the stem chlamyphorid branch (range from 45.45–44.73 Ma). Inactivation dates for *Lipotes* and the stem Delphinidae + Phocoenidae + Monodontidae branch were not calculated because dN/dS values for both branches always exceeded the estimates for pseudogenic branches. However, the elevated dN/dS values for these branches suggest that selection on *ODAM* was relaxed near the base of each branch or even earlier (see Discussion). The mean inactivation date for *Callithrix ODAM* is 13.12 Ma (range = 14.47–11.17 Ma).

**Table 3:** Estimated inactivation times, in millions of years, for select branches where the *ODAM* gene accumulated its first inactivating mutation

Branch	CF1				CF2				Mean inactivation date
	ω_p_ = 1.0 (fixed)		ω_p_ = 1.0446 (estimated)		ω_p_ = 1.0 (fixed)		ω_p_ = 1.106 (estimated)		
	1 syn rate	2 syn rates	1 syn rate	2 syn rates	1 syn rate	2 syn rates	1 syn rate	2 syn rates	
*Orycteropus*	58.99	53.76	54.24	48.26	65.8	62.2	53.91	47.89	55.63
*Dasypus*	41.96	40.63	38.59	36.24	45.45^a^	45.45 ^a^	38.7	36.38	40.43
Stem Chlamyphoridae	45.45^a^	45.45^a^	44.93	44.73	45.45^a^	45.45 ^a^	45.43	45.42	45.29
*Physeter*	26.07	24.04	23.97	21.56	32.43^a^	32.43^a^	26.95	25.14	26.57
Stem Pholidota	65.39	62.60	61.16	57.34	73.04	72.93	62.16	58.55	64.15
*Callithrix*	14.03	12.41	12.90	11.17	15.81	14.47	12.95	11.23	13.12

^a^Estimated inactivation age constrained by divergence time on timetree

Abbreviations: *CF1* codon frequency model 1, *CF2* codon frequency model 2, *syn* synonymous, ω_p_ =dN/dS for pseudogenic branch category

## Discussion

### Patterns of ODAM inactivation in placental mammals

Previous studies have documented inactivating mutations in nine different tooth-related genes in toothless and enamelless mammals (*ACP4, AMBN, AMEL, AMTN, C4orf26, DSPP, ENAM, KLK4, MMP20*). *ODAM* can now be added to this list based on inactivating mutations in both edentulous (Vermilingua, Pholidota, Mysticeti) and enamelless (*Orycteropus*, Folivora, Cingulata) clades. Indeed, inactivating mutations were discovered in all 19 edentulous and enamelless species that were investigated. Given the broad phylogenetic spread of mammalian lineages with inactivating mutations, these results suggest that the only essential functions of *ODAM* that are maintained by natural selection are related to tooth development and/or maintenance of the adhesion of the junctional epithelium to the tooth surface, even though *ODAM* expression has been reported in other tissues such as salivary gland, trachea, mammary gland, and lacrimal gland [14, 15]. The expression of *ODAM* in mammary and salivary glands may be explained by the location of this gene in the same gene expression neighborhood [73] as other SCPP genes that are expressed in mammary glands (*CSN2, CSN3*) and salivary glands (*STATH*).

Patterns of *ODAM* inactivation are generally consistent with ancestral reconstructions of tooth and enamel loss and previous studies of other tooth-related genes [19, 23, 24, 28, 30]. Our estimates for *ODAM* inactivation in Pholidota (73.04–57.34 Ma) are slightly older than Meredith et al.’s [23] estimates for *ENAM* inactivation (59.4–54.9) in this lineage. We recalculated inactivation dates for pangolin *ENAM* using the same divergence times and codon frequency models that were used for *ODAM* and the mean inactivation date is 57.7 Ma (Fig. 5). Estimates for *ODAM* and *ENAM* inactivation are both older than the oldest fossil pangolin, *Eomanis waldi*, which is ~ 47 Ma [23, 74], and suggest that even older fossil pholidotans that lack teeth or at least tooth enamel may be discovered.

Inactivation dates for *ODAM* are also older than inactivation dates for *ENAM* in the aardvark *Orycteropus afer*. Meredith et al. [23] estimated an inactivation date of 35.3–28.8 Ma for *O. afer ENAM*, and we obtained a similar estimate (~ 30.7 Ma) when we used the same divergence dates and codon frequency models as for *ODAM* (Fig. 5).

In Xenarthra, ancestral reconstructions of the presence or absence of enamel suggest that enamel was lost on the stem Pilosa branch [23]. This prediction is validated by two inactivating mutations in *ENAM* that are shared by Vermilingua (anteaters) and Folivora (sloths). In *ODAM*, we discovered inactivating mutations that are shared by the two vermilinguans included in our study, as well as a mutation that is shared by both folivorans, but did not find any mutations that are shared by all pilosans. The dN/dS value for the stem Pilosa branch (~ 0.12–0.14) is below the background dN/dS ratio of ~ 0.49–0.52 and suggests that *ODAM* was maintained by natural selection up until the split between Vermilingua and Folivora. We also re-ran codeml analyses with an added category for the stem Vermilingua and stem Folivora branches, which are the immediate descendant branches of stem Pilosa. The resulting dN/dS values (~ 1.12 [CF1], ~ 1.22 [CF2]) are slightly above the dN/dS values for the pseudogenic branch category (~ 1.04 [CF1], ~ 1.11 [CF2]) and suggest that *ODAM* has evolved neutrally on these branches. Together, the dN/dS values for stem Pilosa, stem Vermilingua, and stem Folivora suggest that selection on *ODAM* was relaxed very near the most recent common ancestor of Pilosa. By contrast, dN/dS analyses for *ENAM* suggest that selection was relaxed near the base of the stem pilosan branch at ~ 64.8 Ma (Fig. 5).

Also in Xenarthra, our estimates of inactivation times in Cingulata (armadillos) suggest that selection on *ODAM* was relaxed very soon after the split between Dasypodidae and Chlamyphoridae at 45.5 Ma [45]. Specifically, selection on *ODAM* was relaxed ~ 40 Ma in *Dasypus* and ~ 45 Ma in stem Chlamyphoridae. Meredith et al. [23] reported inactivating mutations in *ENAM* for four cingulatan genera that they investigated, but none that were shared by all chlamyphorids. In the case of *ENAM*, we estimated an inactivation date of ~ 45.5 Ma for this gene in *Dasypus*. By contrast with pilosans, where inactivating mutations in *ENAM* preceded inactivating mutations in *ODAM*, the opposite pattern occurs in chlamyphorids with an inactivating mutation in *ODAM* preceding the first inactivating mutations in *ENAM* (or at least the region of *ENAM* that was targeted by Meredith et al. [23]).

Whereas *ENAM* is required for enamel formation, *ODAM* inactivation in mouse only causes abnormalities in the junctional epithelium [13]. Thus, the more ancient loss of *ODAM* compared with *ENAM* in both Pholidota and *Orycteropus afer* suggests that selection on maintenance of the junctional epithelium was relaxed before enamel loss likely happened. The inactivation of *ODAM* but not *ENAM* in toothed whales that retain enamel also supports this hypothesis. However, Pilosa and *Dasypus novemcinctus* both show the opposite pattern where inactivation dates for *ENAM* are older than for *ODAM* (Fig. 5).

Beyond its inactivation in toothless and enamelless forms, we also discovered inactivating mutations or exon deletions in taxa with enamel-capped teeth. Two of these (odontocetes, some proboscideans) are discussed in separate sections below. Among the remaining taxa with mutations in *ODAM*, the phocid *Neomonachus* has four separate mutations (2 bp frameshift deletion, premature stop codon, splice site mutation, exon 5 deletion) and is the best candidate for inactivation of this gene among taxa with enamel-capped teeth. However, short read data for *Neomonachus* are not available in the SRA and we were unable to validate these mutations. Another phocid, *Leptonychotes weddellii*, also has problems with the annotated gene sequence, and like *Neomonachus* is missing exon 5 (as well as exons 6–8). SRA data are available for *Leptonychotes* and we were able to reconstruct all of the missing exons for this taxon. It remains unclear if the missing exon and other problems with *Neomonachus ODAM* are real or instead are assembly or annotation errors. *Enhydra lutris* has a 1 bp frameshift in exon 1 of the annotated sequence, but three different individuals with SRA data have both intact and frameshifted versions of this exonic region, which leaves open the possibility that the mutated form of exon 1 represents a second allele, or more likely, a paralogous locus. *Callithrix jacchus* (common marmoset) has an acceptor splice site mutation in intron 4, a premature stop codon in exon 5, and an elevated dN/dS ratio (~ 0.85–0.90). Curiously, marmosets exhibit a loss of lingual enamel and a hypertrophy of buccal enamel in their mandibular incisors [75]. However, similar phenotypes are found in the aye-aye (*Daubentonia madagascariensis*) and rodents, yet they uniformly retain an intact *ODAM*.

### ODAM inactivation in odontocetes

It is perhaps surprising that *ODAM* contains inactivating mutations in all of the odontocetes that we investigated given that all of these taxa retain teeth with enamel. We hypothesize that *ODAM* inactivation in toothed whales may be related to the simplified outer enamel of their teeth. Living odontocetes are characterized by degenerative enamel that is thinner and less complex than the enamel of their archaeocete (stem cetacean) relatives [76–78]. In archaeocetes that have been investigated, there is an inner layer of enamel that is organized into Hunter-Schreger bands (HSB), which are decussating layers of prisms that increase the strength of enamel [79], and an outer layer of radial enamel. This pattern is common in large-bodied, terrestrial mammals and is thought to be a biomechanical adaptation for food processing and crack resistance [78]. By contrast, living odontocetes often swallow prey whole, without mastication, and have variable enamel structure with loss of the HSB in the inner layer. Among Delphinida, which includes all of the odontocetes in our study excepting the sperm whale (*Physeter macrocephalus*), the simplified enamel is typically comprised of an inner layer of radial enamel and an outer layer of aprismatic enamel [77, 78], although in some cases the entire enamel layer is aprismatic [76]. The enamel in *Physeter* has been characterized as being comprised of pseudoprisms. The simplified enamel microstructure of living odontocetes is associated with the transition from heterodont to homodont dentition wherein the upper and lower teeth are used to grasp and secure prey but not for mastication [77, 78]. Given that *ODAM* is expressed in the junctional epithelium, where the gingiva is in contact with the outer enamel surface of the tooth [5], perhaps *ODAM* inactivation in odontocetes is related to the simplified outer enamel surface if this surface is not well suited for ODAM-assisted adhesion. Indeed, *ODAM* is also inactivated in placental mammals that lack enamel on their teeth and instead present dentin on the outer surface (aardvarks, sloths, armadillos).

An alternate hypothesis is that *ODAM* inactivation in toothed whales is related to antimicrobial functions of the junctional epithelium that are altered in aquatic habitats. Specifically, the gingival junctional epithelium adheres tightly to the enamel surface of the tooth in terrestrial mammals and presents a first line of defense against invading bacteria [5]. However, the microbiomes of the oral cavity are expected to differ substantially in terrestrial versus aquatic environments, and toothed whales may require a different line of defense against microbes than is required by terrestrial mammals. The coding sequence of *ODAM* is intact in *Trichechus manatus* (West Indian manatee), which is the other fully aquatic mammal with teeth that was included in our analysis, but codeml analyses revealed an elevated dN/dS value on the *Trichechus* branch (CF1 = 1.60, CF2 = 1.89) when this branch was allowed to have its own category. Although these values are > 1 and suggestive of positive selection, there is no significant difference between a free ratio for *Trichechus* versus a dN/dS value of 1.0 for this branch (*p* = 0.33 with CF1, *p* = 0.18 with CF2). Thus, *ODAM* evolution on the *Trichechus* branch is also consistent with neutral evolution, as might be expected if a gene is evolving neutrally even though the first inactivating mutation has not yet been fixed. It is worth noting that manatees have a unique system of tooth replacement, hind molar progression, in which the molars march forward until they are worn down and replaced by new molars that emerge at the posterior end of the tooth row [80].

### ODAM deletion and functionality in Proboscidea?

**Fig. 6:**
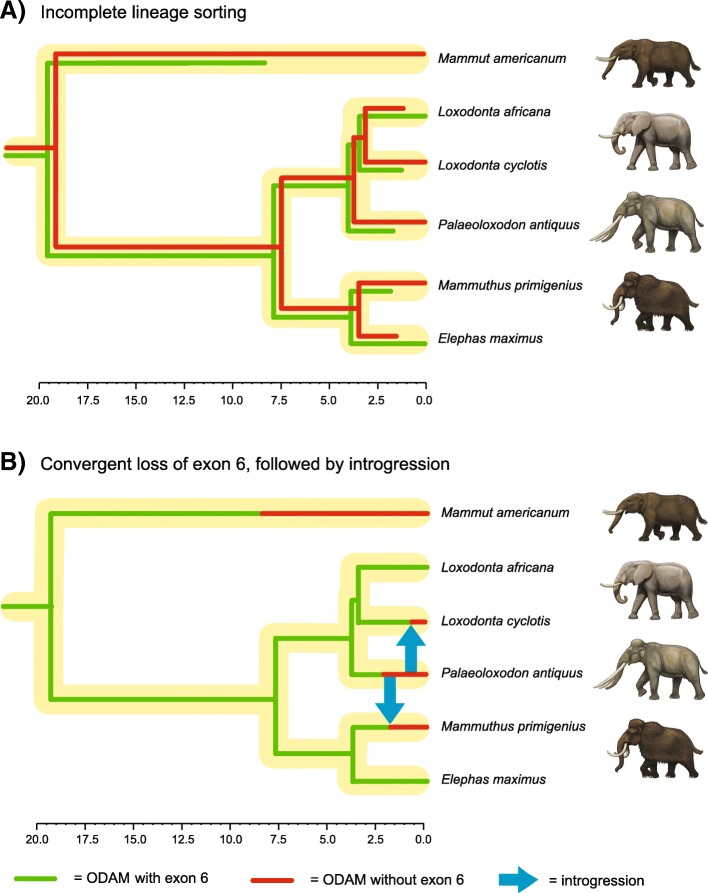
Different hypotheses for the loss of *ODAM* exon 6 in some but not all proboscideans. **a**) Loss of *ODAM* exon 6 based on an ancestral polymorphism in the ancestor of Elephantidae and Mammutidae followed by incomplete lineage sorting of the two allelic variants in Recent and extinct proboscideans. **b**) One possible scenario for the loss of exon 6 of *ODAM* based on convergent loss in *Mammut americanum* and *Palaeoloxodon antiquus* followed by introgression of the sans exon 6 allele from *P. antiquus* to *Loxodonta cyclotis* and *Mammuthus primigenius*. Other scenarios are also possible based on directions of introgression within Elephantidae that were identified by Palkopoulou et al. [71]

Our taxon sampling included six proboscideans, of which five (*Loxodonta africana, L. cyclotis, Elephas maximus, Mammuthus primigenius, Palaeoloxodon antiquus*) belong to Elephantidae and one belongs to Mammutidae (*Mammut americanum*). Exon 6 is putatively missing from four of these taxa based on the absence of sequencing reads that map to this region (Fig. 3). The assembled genome sequence for *L. africana* is also missing exon 6, but we were able to assemble this exon and its flanking intronic regions from SRA data where the Sanger sequencing-based assembly contains a string of Ns (assembly gap). The presence of exon 6 in *L. africana* and *E. maximus*, but not in other proboscideans, is unexpected given the phylogenetic relationships of these taxa. Mammutidae and Elephantidae diverged from each other ~ 30–20 Ma [70, 71], whereas the five elephantids share a much more recent common ancestor ~ 10–5 Ma [71, 81]. Figure 6 shows a time tree for the six proboscideans in our study along with presence/absence data for exon 6 of *ODAM*. Three possible explanations for the presence/absence of exon 6 in these taxa include incomplete lineage sorting (ILS), hybridization, and/or independent deletions of exon 6. Figure 6A shows how ILS can account for the presence/absence of exon 6 in proboscideans, whereas Fig. 6B illustrates how a combination of convergent loss and hybridization can explain the same pattern. Hybridization may play a role in the distribution of *ODAM* exon 6 in Elephantidae given recent evidence for extensive introgression among elephantids [71], but there is no evidence for hybridization between crown elephantids and *Mammut americanum* and this explanation seems unlikely. Instead, ILS or the independent deletion of exon 6 in *Mammut americanum* versus elephantids that lack this exon seems more probable. Is also remains to be determined if individual elephantid species will reveal presence/absence variation for exon 6 when more individuals are sampled, which would not be surprising in view of extensive hybridization among elephantids.

It is also worth noting that the deletion of exon 6 seemingly has an ancient history that may trace as far back as the common ancestor of Elephantidae and Mammutidae if ILS is responsible for the presence/absence of this exon in different proboscideans. Exon 6 is 111 bp in elephantids that have this exon, so complete deletion of this exon will not interrupt the reading frame of the downstream exons (7–10). This leaves open the possibility of an intact version of *ODAM* based on nine of ten exons. The possibility of an altered yet functional version of *ODAM* is also supported by the absence of any frameshift mutations, premature stop codons, or splice site mutations in the remaining nine exons (1–5, 7–10) of the *ODAM* gene of proboscideans that are missing exon 6. Finally, all of the proboscidean *ODAM* sequences that lack exon 6 cluster together on the *ODAM* gene tree and the dN/dS value for this clade is only ~ 0.21–0.23, which is below the background value (~ 0.49–0.52) for branches leading to taxa with taxa with enamel-capped teeth and intact *ODAM* sequences for all ten exons. Thus, beyond the absence of exon 6, there is no evidence for relaxed selection in the *ODAM* gene of proboscideans that lack this exon. Unfortunately a 3D structure for ODAM is not available and it remains unclear if exon 6 encodes a region of the protein that is on the surface or embedded in the interior of this molecule.

### Coevol versus codeml selection analyses

DN/dS analyses with Coevol and codeml were performed with different taxon sets and for different purposes. Coevol was used to implement a joint analysis that included all 165 taxa. This analysis was performed to visualize patterns of dN/dS variation across the placental tree and to examine the effects of life history traits on dN/dS values. Importantly, Coevol employs a form of rate smoothing through the incorporation of a Brownian motion model of continuous trait evolution. By contrast, codeml analyses were performed with 96 taxa and 15 different dN/dS categories that were determined a priori based on ancestral reconstructions of tooth/enamel loss [23, 24] and patterns of *ODAM* inactivation. Coevol and codeml results are generally in agreement with each other with lower dN/dS values for functional branches and higher values for transitional and fully pseudogenic branches. At the same time, dN/dS values for both transitional and pseudogenic branches are generally higher for codeml than for Coevol. For example, Coevol dN/dS values are < 1 for pseudogenic branches in clades that post-date the loss of teeth or enamel. By contrast, dN/dS values for the fully pseudogenic branch category are > 1 in codeml analyses (Table 2). Similarly, the dN/dS value for the transitional branch leading to *Callithrix* is higher for codeml (0.85–0.90) than for Coevol (0.54). We attribute these differences to the autocorrelative or smoothing effect of the Brownian motion model implemented by Coevol [41, 50, 82].

### Is ODAM inactivation neutral or adaptive?

The degeneration of morphological structures such as limbs, teeth, and eyes is a complex process that may result from relaxed selection (neutral evolution), adaptive evolution (direct natural selection to conserve energy and/or eliminate the disadvantageous effects of a morphological structure), and/or pleiotropy (indirect selection on another trait) [83]. Adaptive and non-adaptive causes have also been proposed for the inactivation of different genes [28]. In the case of individual lineages of toothless and enamelless placental mammals, it remains unclear if *ODAM* inactivation resulted from relaxed selection and/or adaptive evolution. For example, was the evolution of edentulism in pangolins driven by the accumulation of random mutations after relaxed selection on tooth development or did natural selection directly favor tooth reduction? If the latter, was this driven by developmental signaling changes that arrested tooth development [84] or were genes that encode structural proteins of the enamel matrix the initial targets of adaptive evolution? This topic is ripe for future studies, but boutique organisms such as pangolins, anteaters, and baleen whales are much less tractable than model organisms such as mice for evo-devo studies of tissue-specific gene expression.

## Conclusions

Nature’s laboratory has provided us with multiple independent lineages of placental mammals that are either toothless or lack enamel caps on their teeth. Molecular evolutionary analysis of candidate tooth-specific genes in these independent lineages has emerged as a powerful approach to test hypotheses that various tooth-related genes are pleiotropic versus tooth-specific with respect to their essential functions that are maintained by natural selection. Previous studies have demonstrated that nine different tooth-related genes are inactivated in one or more clades of mammals that are either edentulous or lack enamel caps on their teeth. Here, we show that *ODAM* is also inactivated in all lineages of toothless and enamelless placental mammals that were investigated. These results support the hypothesis that the only essential functions of *ODAM* that are maintained by natural selection are related to tooth development and the maintenance of a healthy junctional epithelium where the gingivae are in contact with the tooth enamel. Specifically, *ODAM* has been inactivated in all representatives of three toothless clades (Vermilingua, Mysticeti, Pholidota) and three enamelless clades (Folivora, Cingulata, *Orycteropus*) that were included in our study. The overlap in naturally occurring gene inactivations and human genetic diseases [85] suggests that *ODAM* may be linked to some dental or gingival deformities. DN/dS analyses further demonstrate that the *ODAM* gene has evolved neutrally in clades that post-date the occurrence of an inactivating mutation on an earlier branch of the placental tree. DN/dS values for transitional branches, which record the first inactivating mutation in each lineage, also provide the basis for estimating *ODAM* inactivation times. In the main, these estimates are similar to estimates for the inactivation of *ENAM* in these lineages. Estimates of *ENAM* and *ODAM* inactivation in Pholidota both suggest that we may discover stem pholidotans that are older than *Eomanis* (~ 47 Ma) and lack teeth or at least the enamel on their teeth.

Beyond inactivating mutations in toothless and enamelless mammals, we also discovered mutations in several other groups including odontocetes, four proboscideans, and *Callithrix jacchus*. In the case of odontocetes, the inactivation of *ODAM* may be related to a fully aquatic lifestyle where the antimicrobial functions of the junctional epithelium are reduced. An alternate explanation is that the very thin enamel of odontocetes, which is often prismless, may affect the ability of ODAM to contribute to the adhesive properties of the gingival junctional epithelium. The apparent deletion (no BLAST results) of exon 6 of *ODAM* in three elephantids plus *Mammut americanum*, but not in two other elephantids, is unexpected and requires some combination of ILS, hybridization, and/or independent deletions of this exon in different proboscideans. However, proboscideans that lack this exon show no other evidence of inactivating mutations and the remaining nine exons have evolved under purifying selection. These results suggest that a version of *ODAM* that is encoded by nine exons remains functional in these proboscideans.

## Additional files

Additional file 1: **Tables S1.** Source of ODAM sequences for 165 placental mammals. (DOCX 40 kb)

Additional file 2: **Tables S2.** Genes identified in a screen for genes that are preferentially lost in enamelless or edentulous mammals. (XLSX 13.5 kb)

## Abbreviations

bp: Base pairs
ENA: European nucleotide archive
ILS: Incomplete lineage sorting
ODAM: Odontogenic ameloblast-associated
SCPP: Secretory calcium-binding phosphoprotein
SRA: Sequence read archive

## References

[1] Kawasaki K, Buchanan AV, Weiss KM (2009). Biomineralization in humans: making the hard choices in life. Annu Rev Genet.

[2] Kawasaki K (2011). The SCPP gene family and the complexity of hard tissues in vertebrates. Cells Tissues Organs.

[3] Kawasaki K, Weiss KM (2003). Mineralized tissue and vertebrate evolution: the secretory. Proc Natl Acad Sci USA.

[4] Sire J-Y, Davit-Béal T, Delgado S, Gu X (2007). The origin and evolution of enamel mineralization genes. Cells Tissues Organs.

[5] Bosshardt DD, Lang NP (2005). The junctional epithelium: from health to disease. J Dent Res.

[6] Moffatt P, Smith CE, St-Arnaud R, Nanci A (2008). Characterization of Apin, a secreted protein highly expressed in tooth-associated epithelia. J Cell Biochem.

[7] Lee H-K, Lee D-S, Ryoo H-M, Park J-T, Park S-J, Bae H-S, Cho M-I, Park J-C (2010). The odontogenic ameloblast-associated protein (ODAM) cooperates with RUNX2 and modulates enamel mineralization via regulation of MMP-20. J Cell Biochem.

[8] Lee H-K, Ji S, Park S-J, Choung H-W, Choi Y, Lee H-J, Park S-Y, Park J-C. Odontogenic ameloblast-associated protein (ODAM) mediates junctional epithelium attachment to teeth via integrin-ODAM-rho guanine nucleotide exchange factor 5 (ARHGEF5)-RhoA signaling. J Biol Chem. 2015;290:14740–53. 10.1074/jbc.M115.648022 PMC4505539 25911094

[9] Nishio C, Wazen R, Kuroda S, Moffatt P, Nanci A (2010). Expression pattern of odontogenic ameloblast-associated and amelotin during formation and regeneration of the junctional epithelium. Eur Cells Mater..

[10] Fouillen A, Dos Santos Neves J, Charline M, Castonguay J-D, Moffatt P, Baron C, Nanci A (2017). Inactivation of AMTN, ODAM and SCPPPQ1 proteins of a specialized basal lamina that attaches epithelial cells to tooth mineral. Sci Rep.

[11] Park J-C, Park J-T, Son H-H, Kim H-J, Jeong M-J, Lee C-S, Dey R, Cho M-I (2007). The amyloid protein APin is highly expressed during enamel mineralization and maturation in rat incisors. Eur J Oral Sci.

[12] Ikeda Y, Neshatian M, Holcroft J, Ganss B (2018). The enamel protein ODAM promotes mineralization in a collagen matrix. Connect Tissue Res.

[13] Wazen RM, Moffatt P, Ponce KJ, Kuroda S, Nishio C, Nanci A (2015). Inactivation of the odontogenic ameloblast-associated gene affects the integrity of the junctional epithelium and gingival healing. Eur Cells Mater.

[14] Kestler DP, Foster JS, Macy SD, Murphy CL, Weiss DT, Solomon A (2008). Expression of odontogenic ameloblast-associated protein (ODAM) in dental and other epithelial neoplasms. Mol Med.

[15] Lee HK, Park SJ, Oh HJ, Kim JW, Bae HS, Park JC (2012). Expression pattern, subcellular localization, and functional implications of ODAM in ameloblasts, odontoblasts, osteoblasts, and various cancer cells. Gene Expr Patterns.

[16] Sire J-Y, Delgado S, Girondot M (2008). Hen’s teeth with enamel cap: from dream to impossibility. BMC Evol Biol.

[17] Davit-Béal T, Tucker AS, Sire J-Y (2009). Loss of teeth and enamel in tetrapods: fossil record, genetic data and morphological adaptations. J Anat.

[18] Al-Hashimi N, Lafont A-G, Delgado S, Kawasaki K, Sire J-Y (2010). The enamelin genes in lizard, crocodile, and frog and the pseudogene in the chicken provide new insights on enamelin evolution in tetrapods. Mol Biol Evol.

[19] Meredith RW, Zhang G, Gilbert MTP, Jarvis ED, Springer MS (2014). Evidence for a single loss of mineralized teeth in the common avian ancestor. Science.

[20] Meredith RW, Gatesy J, Springer MS (2013). Molecular decay of enamel matrix protein genes in turtles and other edentulous amniotes. BMC Evol Biol.

[21] Deméré TA, McGowen MR, Berta A, Gatesy J (2008). Morphological and molecular evidence for a stepwise evolutionary transition from teeth to baleen in mysticete whales. Syst Biol.

[22] McKnight DA, Fisher LW (2009). Molecular evolution of dentin phosphoprotein among toothed and toothless animals. BMC Evol Biol.

[23] Meredith RW, Gatesy J, Murphy WJ, Ryder OA, Springer MS (2009). Molecular decay of the tooth gene enamelin (*ENAM*) mirrors the loss of enamel in the fossil record of placental mammals. PLoS Genet.

[24] Meredith RW, Gatesy J, Cheng J, Springer MS (2011). Pseudogenization of the tooth gene enamelysin (*MMP20*) in the common ancestor of extant baleen whales. Proc R Soc B.

[25] Kawasaki K, JC-C H, Simmer JP (2014). Evolution of *Klk4* and enamel maturation in eutherians. Biol Chem.

[26] Springer MS, Signore AV, Paijmans JLA, Vélez-Juarbe J, Domning DP, Bauer CE, He K, Crerar L, Campos PF, Murphy WJ, Meredith RW, Gatesy J, Willerslev E, MacPhee RDE, Hofreiter M, Campbell KL (2015). 2015 Interordinal gene capture, the phylogenetic position of Steller’s sea cow based on molecular and morphological data, and the macroevolutionary history of Sirenia. Mol Phylogenet Evol.

[27] Springer MS, Starrett J, Morin PA, Lanzetti A, Hayashi C, Gatesy J (2016). Inactivation of *C4orf26* in toothless placental mammals. Mol Phylogenet Evol.

[28] Sharma V, Hecker N, Roscito JG, Foerster L, Langer BE, Hiller M (2018). A genomics approach reveals insights into the importance of gene losses for mammalian adaptations. Nat Commun.

[29] Gasse B, Silvent J, Sire J-Y (2012). Evolutionary analysis suggests that *AMTN* is enamel-specific and a candidate for AI. J Dent Res.

[30] Delsuc F, Gasse B, Sire J-Y (2015). Evolutionary analysis of selective constraints identifies ameloblastin (AMBN) as a potential candidate for amelogenesis imperfecta. BMC Evol Biol.

[31] Kearse M, Moir R, Wilson A, Stones-Havas S, Cheung M, Sturrock S, Buxton S, Cooper A, Markowitz S, Duran C, Thierer T, Ashton B, Mentjies P, Drummond A (2012). Geneious basic: an integrated and extendable desktop software platform for the organization and analysis of sequence data. Bioinformatics.

[32] Katoh K, Misawa K, Kuma K, Miyata TMAFFT (2002). A novel method for rapid multiple sequence alignment based on fast Fourier transform. Nucleic Acids Res.

[33] Katoh K (2013). Stanley DM. MAFFT multiple sequence alignment software version 7: improvements in performance and usability. Mol Biol Evol.

[34] Ranwez V, Harispe S, Delsuc F, Douzery EJP (2011). MACSE: multiple alignment of coding SEquences accounting for frameshifts and stop codons. PLoS One.

[35] Ranwez V, Douzery EJP, Cambon C, Chantret N, Delsuc F (2018). MACSE v2: toolkit for the alignment of coding sequences accounting for frameshifts and stop codons. Mol Biol Evol.

[36] Swofford DL, PAUP* (2002). Phylogenetic analysis using parsimony (* and other methods).

[37] Stamatakis A (2006). RAxML-VI-HPC: maximum likelihood-based phylogenetic analyses with thousands of taxa and mixed models. Bioinformatics.

[38] Stamatakis A. RAxML version 8: a tool for phylogenetic analysis and post-analysis of large phylogenies. Bioinformatics 201;30;1312–1313. 10.1093/bioinformatics/btu033 PMC3998144 24451623

[39] Miller MA, Pfeiffer W, Schwartz T. Creating the CIPRES science gateway for inference of large phylogenetic trees. Gateway Computing Environments Workshop. 2010:1–8.

[40] Stamatakis A, Hoover P, Rougemont J (2008). A rapid bootstrap algorithm for the RAxML web servers. Syst Biol.

[41] Lartillot N, Poujol R (2010). A phylogenetic model for investigating correlated evolution of substitution rates and continuous phenotypic characters. Mol Biol Evol.

[42] Lartillot N, Delsuc F (2012). Joint reconstruction of divergence times and life-history evolution in placental mammals using a phylogenetic covariance model. Evolution.

[43] Teeling EC, Springer MS, Madsen O, Bates P, O’Brien SJ (2005). Murphy WJ. A molecular phylogeny for bats illuminates biogeography and the fossil record. Science.

[44] Meredith RW, Janecka JE, Gatesy J, Ryder OA, Fisher CA, Teeling EC, Goodbla A, Eizirik E, Simão TLL, Stadler T, Rabosky DL, Honeycutt RL, Flynn JJ, Ingram CM, Steiner C, Williams TL, Robinson TJ, Burk-Herrick A, Westerman M, Ayoub NA, Springer MS, Murphy WJ. Impacts of the Cretaceous Terrestrial Revolution and KPg extinction on mammal diversification. Science. 2011;334:521–4. 10.1126/science.1211028 21940861

[45] Delsuc F, Gibb GC, Kuch M, Billet G, Hautier L, Southon J, Rouillard J-M, Fernicola JC, Vizcaíno SF, MacPhee RDE, Poinar HN (2016). The phylogenetic affinities of the extinct glyptodonts. Curr Biol.

[46] Foley NM, Springer MS, Teeling EC (2016). Mammal madness: is the mammal tree of life not yet resolved?. Philos Trans R Soc B.

[47] Emerling CA, Huynh HT, Nguyen MA, Meredith RW, Springer MS. Spectral shifts of mammalian ultraviolet-sensitive pigments (short wavelength-sensitive opsin 1) are associated with eye length and photic niche evolution. Proc Roy Soc B. 2015;282:20151817. 10.1098/rspb.2015.1817 PMC4685808 26582021

[48] Jones KE, Bielby J, Cardillo M, Fritz SA, O'Dell J, Orme CDL, Safi K, Sechrest W, Boakes EH, Carbone C, Connolly C, Cutts MJ, Foster JK, Grenyer R, Habib M, Plaster CA, Price SA, Rigby EA, Rist J, Teacher A, Bininda-Emonds ORP, Gittleman JL, Mace GM, Purvis A (2009). PanTHERIA: a species-level database of life history, ecology, and geography of extant and recently extinct mammals. Ecology.

[49] Yang Z (2007). PAML 4: phylogenetic analysis by maximum likelihood. Mol Biol Evol.

[50] Thorne JL, Kishono H, Painter IS (1998). Estimating the rate of evolution of the rate of molecular evolution. Mol Biol Evol.

[51] Yeo G, Burge CB (2004). Maximum entropy modeling of short sequence motifs with applications to RNA splicing signals. J Comp Biol.

[52] Nitsche A, Rose D, Fasold M, Reiche K, Stadler PF (2015). Comparison of splice sites reveals that long noncoding RNAs are evolutionarily well conserved. RNA.

[53] Springer MS, Meredith RW, Gatesy J, Emerling CA, Park J, Rabosky DL, Stadler T, Steiner C, Ryder OA, Janecka JE, Fisher CA, Murphy WJ (2012). Macroevolutionary dynamics and historical biogeography of primate diversification inferred from a species supermatrix. PLoS One.

[54] Gaubert P, Antunes A, Meng H, Miao L, Peigné S, Justy F, Njiokou F, Dufour S, Danquah E, Alahakoon J, Verheyen E (2017). The complete phylogeny of pangolins: scaling up resources for the molecular tracing of the most trafficked mammals on earth. J Heredity.

[55] Prudent X, Parra G, Schwede P, Roscito JG, Hiller M (2016). Controlling for phylogenetic relatedness and evolutionary rates improves the discovery of associations between species’ phenotypic and genomic differences. Mol Biol Evol.

[56] Madsen O, Scally M, Douady CJ, Kao DJ, DeBry RW, Adkins R, Amrine HM, Stanhope MJ, de Jong WW, Springer MS (2001). Parallel adaptive radiations in two major clades of placental mammals. Nature.

[57] Murphy WJ, Eizirik E, Johnson WE, Zhang YP, Ryder OA, O’Brien SJ (2001). Molecular phylogenetics and the origins of placental mammals. Nature.

[58] Murphy WJ, Eizirik E, O’Brien SJ, Madsen O, Scally M, Douady CJ, Teeling E, Ryder OA, Stanhope MJ, de Jong WW, Springer MS (2001). Resolution of the early placental mammal radiation using Bayesian phylogenetics. Science.

[59] Scally M, Madsen O, Douady CJ, de Jong WW, Stanhope MJ, Springer MS. Molecular evidence for the major clades of placental mammals. J Mammal Evol 2001;8:239–277. 10.1038/35054544 11214318

[60] Waddell PJ, Kishino H (2001). Ota R. A phylogenetic foundation for comparative mammalian genomics. Genome Informatics.

[61] Delsuc F, Scally M, Madsen O, Stanhope MJ, de Jong W, Catzeflis FM, Springer MS, Douzery EJP (2002). Molecular phylogeny of living xenarthrans and the impact of character and taxon sampling on the placental tree rooting. Mol Biol Evol.

[62] Springer MS, Murphy WJ, Eizirik E, O’Brien SJ. Placental mammal diversification and the Cretaceous-Tertiary boundary. Proc Natl Acad Sci U S A. 2003;100:1056–61. 10.1073/pnas.0334222100 PMC298725 12552136

[63] Springer MS, Stanhope MJ, Madsen O, de Jong WW (2004). Molecules consolidate the placental mammal tree. Trends Ecol Evol.

[64] Springer MS, Murphy WJ, Eizirik E, O’Brien SJ, Rose KD, Archibald JK (2005). Evidence for major placental clades. The rise of placental mammals: origins and relationships of the major extant clades.

[65] McGowen MR, Spaulding M, Gatesy J (2009). Divergence date estimation and a comprehensive molecular tree of extant cetaceans. Mol Phylogenet Evol.

[66] Gatesy J, Geisler JH, Chang J, Buell C, Berta A, Meredith RW, Springer MS, McGowen MR (2013). A phylogenetic blueprint for a modern whale. Mol Phylogenet Evol.

[67] Teeling EC, Madsen O, Van Den Bussche RA, de Jong WW, Stanhope MJ, Springer MS (2002). Microbat paraphyly and the convergent evolution of a key innovation in Old World rhinolophoid microbats. Proc Natl Acad Sci U S A.

[68] Roca AL, Bar-Gal GK, Eizirik E, Helgen KM, Maria R, Springer MS, O’Brien SJ, Murphy WJ. Mesozoic origin for West Indian insectivores. Nature. 2004;429:649–51. 10.1038/nature02597 15190349

[69] Springer MS, Murphy WJ, Roca AL (2018). Appropriate fossil calibrations and tree constraints uphold the Mesozoic divergence of solenodons from other extant mammals. Mol Phylogenet Evol.

[70] Shoshani J, Walter RC, Abraha M, Berhe S, Tassy P, Sanders WJ, Marchant GH, Libeskal Y, Ghirmai T, Zinner D (2006). A proboscidean from the late Oligocene of Eritrea, a “missing link” between early Elephantiformes and Elephantimorpha, and biogeographic implications. Proc Natl Acad Sci U S A.

[71] Palkopoulou E, Lipson M, Mallick S, Nielsen S, Rohland N, Baleka N, Karpinski S, Ivancevic AM, Kortschak RD, Raison JM, Qu Z, Chin T-J, Alt KW, Claesson S, Dalén L, MacPhee RDE, Meller H, Roca AL, Ryder OA, Heiman D, Young S, Breen M, Williams C, Aken BL, Ruffier M, Karlsson E, Johnson J, Di Palma F, Alfordi J, Adelson DL, Mailund T, Munch K, Lindblad-Toh K, Hofreiter M, Poiner H, Reich D, To T-H (2018). A comprehensive genomic history of extinct and living elephants. Proc Natl Acad Sci U S A.

[72] Sharma V, Elghafari A, Hiller M (2016). Coding exon-structure aware realigner (CESAR) utilizes genome alignments for accurate comparative gene annotation. Nucleic Acids Res.

[73] Oliver B, Parisi M, Clark D (2002). Gene expression neighborhoods. J Biol.

[74] Franzen JL (2005). The implications of the numerical dating of the Messel fossil deposit (Eocene, Germany) for mammalian biochronology. Ann Paleontol.

[75] Rosenberger AL (1978). Loss of incisor enamel in marmosets. J Mammal.

[76] Ishiyama M (1987). Enamel structure in odontocete whales. Scan Microsc.

[77] Loch C, Duncan W, Simões-Lopes PC, Kieser JA, Fordyce RE (2013). Ultrastructure of enamel and dentine in extant dolphins (Cetacea: Delphinoidea and Inioidea). Zoomorphology.

[78] Loch C, Kieser JA, Fordyce RE (2015). Enamel ultrastructure in fossil cetaceans (Cetacea: Archaeoceti and Odontoceti). PLoS One.

[79] Koenigswald W, Rensberger JM, Pretzschner HU (1987). Changes in the tooth enamel of early Paleocene mammals allowing increased diet diversity. Nature.

[80] Domning DP (1983). Marching teeth of the manatee. Nat Hist.

[81] Rohland N, Reich D, Mallick S, Meyer M, Green RE, Georgiadis NJ, Roca AL, Hofreiter M (2010). Genomic DNA sequences from mastodon and woolly mammoth reveal deep speciation of forest and savanna elephants. PLoS Biol.

[82] Seo TK, Kishino H, Thorne JL (2004). Estimating absolute rates of synonymous and nonsynonymous nucleotide substitution in order to characterize natural selection and date species divergences. Mol Biol Evol.

[83] Krishnan J, Rohner N (2017). Cavefish and the basis for eye loss. Philos Trans Roy Soc B.

[84] Tokita M, Chaeychomsri W, Siruntawineti J (2013). Developmental basis of toothlessness in turtles: insight into convergent evolution of vertebrate morphology. Evolution.

[85] Emerling CA, Widjaja AD, Nguyen NN, Springer MS (2017). Their loss is our gain: regressive evolution in vertebrates provides genomic models for uncovering human disease loci. J Med Genet.

