# Supplementary material for: Odontogenic ameloblast-associated (ODAM) is inactivated in toothless/enamelless placental mammals and toothed whales
Source: BMC Evol Biol. 2019 Jan 23;19:31. doi: 10.1186/s12862-019-1359-6 (PMC6343362; doi:10.1186/s12862-019-1359-6)
Supplement: Supplementary file 1 — Tables S1. Source of ODAM sequences for 165 placental mammals. (DOCX 40 kb) [file 12862_2019_1359_MOESM1_ESM.docx]

Table S1. Source of *ODAM* sequences for 165 placental mammals.

| Taxon | Assembled genome (AG), unassembled genome data from SRA or ENA data (UG), or unpublished Illumina data (UID) | Accession number(s) |
| --- | --- | --- |
| 1. **Order Proboscidea** |  |  |
| *Elephas maximus* | UG | SRX1015604 |
| *Loxodonta africana* | AG (exons 1-5, 7-10); UG (exon 6) | NW_003573450 (exons 1-5, 7-10); SRR958467 and SRR9058468 (exon 6) |
| *Loxodonta cyclotis* | UG | ERR2260495 |
| *Mammuthus primigenius* | UG | SRX3070749 |
| *Palaeoloxodon antiquus*^1^ | UG | ERR2260504 |
| *Mammut americanum* | UG | ERR2260503 |
| 1. **Order Sirenia** |  |  |
| *Trichechus manatus* | AG | NW_004444044 |
| 1. **Order Hyracoidea** |  |  |
| *Procavia capensis* | AG | ABRQ02044779 |
| 1. **Order Tubulidentata** |  |  |
| *Orycteropus afer* | AG | NW_006921685 |
| 1. **Order Afrosoricida** |  |  |
| *Chrysochloris asiatica* | AG | NW_006408772 |
| *Echinops telfairi* | AG | NW_004558715 |
| 1. **Order Macroscelidea** |  |  |
| *Elephantulus edwardii* | AG | NW_006399942 |
| 1. **Order Pilosa** |  |  |
| *Cyclopes didactylus* | UID | MK344314 |
| *Tamandua tetradactyla* | UID | MK344315 |
| *Choloepus didactylus* | UID | MK344313 |
| *Choloepus hoffmanni* | AG | ABVD02348737 |
| 1. **Order Cingulata** |  |  |
| *Dasypus novemcinctus* | AG | NW_004491660 |
| *Tolypeutes matacus* | UID | MK344316 |
| *Cabassous unicinctus* | UID | MK344309 |
| *Chaetophractus vellerosus* | UID | MK344311 |
| *Calyptophractus retusus* | UID | MK344310 |
| *Chlamyphorus truncatus* | UID | MK344312 |
| 1. **Order Rodentia** |  |  |
| *Spermophilus tridecemlineatus* | AG | NW_004936598 |
| *Marmota marmot* | AG | NW_015351313 |
| *Octogon degus* | AG | NW_004524917 |
| *Cavia porcellus* | AG | NT_176372 |
| *Cavia aperea* | AG | AVPZ01000422 |
| *Chinchilla lanigera* | AG | NW_004955447 |
| *Tympanoctomys barrerae* | AG | NDGN011462117 (exons 1-5); NDGN011269361 (exons 6-8); NDGN011546706 (exons 9-10) |
| *Octomys mimax* | AG | NDGM010253829 (exons 1-5); NDGM010984016 (exon 6); NDGM010795039 (exons 7-8); NDGM01120814 (exons 9-10) |
| *Fukomys damarensis* | AG | NW_011046557 |
| *Heterocephalus glaber* | AG | NW_004624890 |
| *Dipodomys ordii* | AG | NW_012267265 |
| *Castor canadensis* | AG | NW_017878596 |
| *Jaculus jaculus* | AG | NW_004504390 |
| *Nannospalax galili* | AG | NW_008328749 |
| *Meriones unguiculatus* | AG | NW_018666917 |
| *Mesocricetus auratus* | AG | NW_004801616 |
| *Phodopus sungorus* | AG | MCBN011461350 (exons 1-3); MCBN011093958 (exons 4-5); MCBN011325814 (exons 6-8); MCBN010560716 (exons 9-10) |
| *Cricetulus griseus* | AG | NW_006879716 |
| *Ellobius lutescens* | AG | LOJG01008886 |
| *Ellobius talpinus* | AG | LOJH01038791 |
| *Microtus agrestis* | AG | LIQJ01009635 |
| *Microtus ochrogaster* | AG | NC_022027 |
| *Peromyscus maniculatus* | AG | NW_006501573 |
| *Myodes glareolus* | AG (exons 1-3, 6-8); UG (exons 4-5, 9-10) | LIPI01017564 (exons 1-3, 6-8); SRR4342176 + SRR4449962 (exons 4-5, 9-10) |
| *Mus caroli* | AG | NC_034574 |
| *Mus parahi* | AG | NC_034602 |
| *Mus musculus* | AG | NC_000071 |
| *Rattus norvegicus* | AG | NC_005113 |
| 1. **Order Lagomorpha** |  |  |
| *Ochotona princeps* | AG | NW_004535515 |
| *Oryctolagus cuniculus* | AG | NC_013683 |
| 1. **Order Primates** |  |  |
| *Daubentonia madagascariensis* | AG | AGTM011616431 |
| *Microcebus murinus* | AG | NC_033685 |
| *Eulemur macaco* | AG | LGHX01003957 |
| *Eulemur flavifrons* | AG | LGHW01004004 |
| *Propithecus coquereli* | AG (exons 3-7); UG (exons 1-2, 8-10) | JZKE01245378 (exons 3-5); JZKE01097126 (exon 6); JZKE01245379 (exon 7); SRR1575542 (exons 1-2, 8-10) |
| *Otolemur garnettii* | AG | AAQR03168055 |
| *Tarsius syrichta* | AG | ABRT02438883 |
| *Callithrix jacchus* | AG | NC_013898 |
| *Aotus nancymaae* | AG | NW_018503765 |
| *Cebus capucinus* | AG | LVWQ01085801 |
| *Saimiri boliviensis* | AG | NW_003943651 |
| *Nasalis larvatus* | AG | JMHX01319533 |
| *Colobus angolensis* | AG | NW_012120710 |
| *Piliocolobus tephrosceles* | AG | NW_019318474 |
| *Rhinopithecus roxellana* | AG | NW_010790249 |
| *Rhinopithecus bieti* | AG | NW_016803348 |
| *Cercocebus atys* | AG | NW_012001894 |
| *Papio anubis* | AG | NC_018156 |
| *Mandrillus leucophaeus* | AG | NW_012109108 |
| *Chlorocebus sabaeus* | AG | NC_023648 |
| *Macaca fascicularis* | AG | NC_022276 |
| *Macaca mulatta* | AG | NC_027897 |
| *Macaca nemestrina* | AG | NW_012016799 |
| *Homo sapiens* | AG | NC_000004 |
| *Pan troglodytes* | AG | NC_006471 |
| *Pan paniscus* | AG | NC_027872 |
| *Gorilla gorilla* | AG | NC_018428 |
| *Pongo abelii* | AG | NC_012595 |
| *Nomascus leucogenys* | AG | NC_019824 |
| 1. **Order Dermoptera** |  |  |
| *Galeopterus variegatus* | AG | NW_007728571 |
| 1. **Order Scandentia** |  |  |
| *Tupaia belangeri* | AG | NW_006159548 |
| 1. **Order Eulipotyphla** |  |  |
| *Condylura cristata* | AG | NW_004567113 |
| *Sorex araneus* | AG | NW_004546063 |
| *Suncus murinus* | UG | DRX024926 |
| *Erinaceus europaeus* | AG | NW_006804129 |
| *Solenodon paradoxus* | AG | NKTL01000704 |
| 1. **Order Chiroptera** |  |  |
| *Rousettus aegyptiacus* | AG | NW_015494973 |
| *Pteropus vampyrus* | AG | NW_011888837 |
| *Pteropus alecto* | AG | NW_006440624 |
| *Eidolon helvum* | AG | AWHC01266858 (exons 1-8); AWHC01287588 (exons 9-10) |
| *Megaderma lyra* | AG | AWHB01142960 (exons 1-5);  AWHB01409051 (exons 6-10) |
| *Rhinolophus ferrumequinum* | AG | AWHA01187516 |
| *Rhinolophus sinicus* | AG | NW_017739384 |
| *Hipposideros armiger* | AG | NW_017731736 |
| *Eptesicus fuscus* | AG | NW_007370817 |
| *Myotis brandtii* | AG | NW_005370660 |
| *Myotis lucifugus* | AG | NW_005871497 |
| *Myotis davidii* | AG | NW_006297290 |
| *Miniopterus natalensis* | AG | NW_015504408 |
| *Pteronotus parnellii* | AG | AWGZ01411607 |
| 1. **Order Carnivora** |  |  |
| *Panthera pardus* | AG | NW_017619847 |
| *Panthera tigris* | AG | NW_006711939 |
| *Panthera leo* | UG | SRR836361 |
| *Felis catus* | AG | NC_018726 |
| *Acinonyx jubatus* | AG | NW_015130656 |
| *Hyaena hyaena* | AG | PEQU01000398 |
| *Lycaon pictus* | AG | LPRA01000013 |
| *Canis lupus* | AG | NC_006595 |
| *Ailuropoda melanoleuca* | AG | LNAT01000059 |
| *Ursus maritimus* | AG | AVOR01041391 |
| *Ailurus fulgens* | AG | LNAC01000013 |
| *Neovison vison* | AG | FNWR01000089 |
| *Mustela putorius* | AG | NW_004569262 |
| *Enhydra lutris* | AG | NSES01008467 |
| *Arctocephalus gazella* | UG | SRR2658571 + SRR2658582 + SRR2658583 |
| *Neomonachus schauinslandi* | AG | NW_018734389 |
| *Leptonychotes weddellii* | AG (exons 1-4, 9-10); UG (exons 5-8) | NW_006383182 (exons 1-4, 9-10); SRR317817 + SRR317818 + SRR317810 + SRR317820 (exons 5-8) |
| *Odobenus rosmarus* | AG | NW_004450275 |
| 1. **Order Pholidota** |  |  |
| *Manis javanica* | AG | NW_016534235 (exons 1-5); NW_016545962 (exons 6-10) |
| *Manis pentadactyla* | UG | SRR2591032 |
| 1. **Order Cetartiodactyla** |  |  |
| *Camelus bactrianus* | AG | NW_011517196 |
| *Camelus ferus* | AG | NW_006211284 |
| *Camelus dromedarius* | AG | NW_011591251 |
| *Vicugna pacos* | AG | NW_005882856 |
| *Sus scrofa* | AG | NC_010450 |
| *Bubalus bubalis* | AG (exons 1-6, 9-10); UG (exons 7-8) | NW_005785429 (exons 1-6, 9-10); SRR032564 + SRR034148 (exons 7-8) |
| *Bison bison* | AG (exons 1-8); UG (exons 9-10) | JPYT01271772 (exons 1-8); SRR3530515 (exons 9-10) |
| *Bos taurus* | AG | AAFC05011017 |
| *Bos indicus* | AG | AGFL01059868 |
| *Bos mutus* | AG (exons 1-8); UG (exons 9-10) | AGSK01123287 (exons 1-8); SRR361209 (exons 9-10) |
| *Ammotragus lervia* | AG (exons 1-8); UG (exons 9-10) | NIVO01024987 (exons 1-8); SRR5438049 (exons 9-10) |
| *Ovis aries* | AG | AMGL02018607 |
| *Capra hircus* | AG | LWLT01000006 |
| *Capra aegagrus* | AG | CBYH010035622 |
| *Pantholops hodgsonii* | AG | NW_005817850 |
| *Capreolus capreolus* | AG | CCMK010072099 |
| *Elaphurus davidianus* | AG | JRFZ0112955 |
| *Odocoileus virginianus* | AG | NW_018338687 |
| *Cervus elaphus* | AG | MKHE01000006 |
| *Giraffa camelopardalis* | AG | LVKQ01150418 (exons 1-3); LVKQ01150417 (exons 4-5);  LVKQ01150416 (exons 6-10) |
| *Okapia johnstoni* | AG | LVCL010177723 |
| *Hippopotamus amphibius* | AG | NKPW01007035 |
| *Choeropsis liberiensis* | UID | MK353459 |
| *Eschrichtius robustus* | AG | NIPP01012926 |
| *Balaenoptera acutorostrata* | AG | NW_006732456 |
| *Balaenoptera bonaerensis* | AG (exons 1-8); UG (exons 9-10) | BAUQ01140012 (exons 1-3); BAUQ01140011 (exons 4-5); BAUQ01140010 (exons 6-8);  SRR4011113 (exons 9-10) |
| *Balaenoptera borealis* | UG | SRR5665645 |
| *Balaenoptera musculus* | UG | SRR5665644 |
| *Balaena mysticetus* | AG | Scaffold 1915^2^ |
| *Eubalaena glacialis* | UG | SRR5665640 |
| *Physeter macrocephalus* | AG | NW_006716159 |
| *Delphinapterus leucas* | AG | NW_019160980 |
| *Phocoena phocoena* | AG | PKGA01135801 |
| *Neophocaena asiaeorientalis* | AG | MKKW01012928 |
| *Neophocaena phocaenoides* | UG | SRR940959 |
| *Tursiops truncatus* | AG | NW_017842696 |
| *Orcinus orca* | AG | NW_004438441 |
| *Lipotes vexillifer* | AG | NW_006775759 |
| 1. **Order Perissodactyla** |  |  |
| *Equus asinus* | AG | NW_014637991 |
| *Equus caballus* | AG | PJAA01000004 |
| *Equus przewalskii* | AG | NW_007677999 |
| *Dicerorhinus sumatrensis* | AG | PEKH010001114 |
| *Ceratotherium simum* | AG | NW_004454157 |

^1^Same as *Elephas antiquus*.

^2^ http://www.bowhead-whale.org/
